# Leveraging Microbe–Rhizosphere Interactions in Organic Farming Systems: A Route to Sustainable Soybean Production

**DOI:** 10.1002/pei3.70147

**Published:** 2026-04-09

**Authors:** Ijeoma Emelda Osuji, Akinlolu Olalekan Akanmu, Olubukola Oluranti Babalola

**Affiliations:** ^1^ Food Security and Safety Focus Area, Faculty of Natural and Agricultural Sciences North‐West University Mmabatho South Africa; ^2^ Department of Life Sciences Imperial College London Berkshire UK

**Keywords:** microbial diversity, organic farming systems, plant‐microbe interactions, soybean rhizosphere, sustainable agriculture

## Abstract

Soybean ( *Glycine max* L.) is a major legume crop of global agricultural significance, and its yield is heavily dependent on the rhizospheric microbes. Conventional farming systems can enhance yields in the short term but often at the expense of soil health and biodiversity. Organic farming systems, by contrast, avoid the use of synthetic inputs and depend on microbial processes to achieve yield. This review aggregates peer‐reviewed literature on organic soybean farming systems, drawing from a body of work that has characterized the diversity, composition, and functions of rhizospheric microbes in these systems. Organic amendments such as compost, manure, and biochar enhance the abundance of microbial communities in the rhizosphere of organic soybean crops, buffer soil pH, and improve soil structure. Organic soils have greater microbial biomass and functional activity than conventional soils, with increased populations of bacteria such as *Bradyrhizobium*, arbuscular mycorrhizal fungi, *Trichoderma, Streptomyces*, and phosphate‐solubilizing bacteria. The rhizospheric microbes are responsible for processes such as nitrogen fixation, phosphorus acquisition, organic matter decomposition, and induced systemic resistance (ISR). Measures of soil health, such as microbial biomass, enzyme activity, respiration rates, and soil organic matter (SOM) content, all demonstrate that organic farming systems have greater ecological value than conventional systems. Organic soybean production systems foster distinct rhizosphere microbial assemblages that confer measurable functional benefits to the agroecosystem. Future research is required in microbiome engineering, biostimulant design for specific applications, biomarkers for monitoring changes in soil microbiology, and precision organic farming systems. This review demonstrates that microbe–rhizosphere interactions are a key factor to consider in the development of sustainable agricultural practices for soybean production.

## 1 Introduction

Soybean ( *Glycine max* L.) is one of the most economically important legumes globally. It serves as a key source of protein and edible oil, provides livestock feed, and supports the production of various industrial products. Beyond its economic significance, the soybean is also an important ecological crop that supports biological nitrogen fixation (BNF) in symbiosis with rhizobia. This process converts atmospheric N_2_ into ammonia used for plant growth and development. It also reduces the need for nitrogenous fertilizers and maintains the nitrogen balance of the agroecosystem. However, soybean and other crops are becoming increasingly challenged by factors such as soil degradation, loss of biodiversity, greenhouse gas emissions, and climate change associated with intensive conventional farming practices (Agyekum et al. 2023; Oliveira et al. 2024). These challenges have led to a significant increase in the adoption of organic farming systems as a biologically based alternative farming system that restores soil health, enhances ecosystem services, and reduces the use of synthetic chemicals.

The rhizosphere is the soil region that is dynamically influenced by the activities of plant roots. It is a biochemical and ecological hub through which plant‐derived carbon substrates influence the assembly, gene expression, and function of microorganisms (Dlamini et al. 2022). Root exudates comprising low‐molecular‐weight compounds such as sugars, amino acids, organic acids, phenolics, and diverse secondary metabolites serve as critical chemical signals and substrates that shape microbial recruitment, community assembly, and functional dynamics within the rhizosphere (Liu et al. 2024; Olanrewaju et al. 2019). In soybean farming systems in particular, the composition and function of rhizosphere microbial communities are especially important because of the plant's dependence on *Bradyrhizobium* spp. for BNF. Plant‐derived carbon compounds are used in molecular signaling pathways that establish symbiosis with rhizobia, including flavonoids, *Nod* factors, and quorum‐sensing molecules (Haskett et al. 2022). In addition to host genotype effects, a wide variety of environmental factors can affect the composition of rhizosphere microbial communities. These factors include soil properties, climate conditions, and management practices (Fadiji et al. 2023).

Organic management systems modify both the structure and functional capacity of rhizosphere microbial communities through the application of biologically active amendments and by minimizing disturbances associated with synthetic fertilizers and pesticide inputs. Organic amendments including compost, manure, green manure, and biochar increase soil organic matter (SOM), enhance cation exchange capacity (CEC), and improve aggregate stability and buffer capacity, which promote the development of favorable conditions for microbial growth and diversity (Akanmu et al. 2020; Ajiboye et al. 2022). SOM improves soil physical and chemical properties, creating favorable conditions for microbial growth and diversity. Labile carbon compounds in compost and manure increase the metabolic activity of heterotrophic microorganisms, whereas recalcitrant compounds in biochar support long‐term carbon sequestration and habitat stabilization. Biochar also modifies soil properties and conditions, such as water retention and aeration, which favor the colonization of symbiotic microorganisms like *Bradyrhizobium* and AMF (Fornes et al. 2024). The resulting modifications in soil properties and conditions have been consistently associated with increased microbial biomass carbon (MBC), elevated enzyme activities (e.g., β‐glucosidase, urease, phosphatase), and improved nutrient cycling compared with conventionally managed soils (Uwituze et al. 2022).

High‐throughput sequencing and functional gene analyses have shown that organic management systems increase the richness and evenness of microbial communities in soybean rhizospheres compared with conventional farming systems. The rhizospheres of organic soybean farming systems contain higher levels of phyla such as Proteobacteria, Actinobacteria, and Acidobacteria compared with conventional farming systems (Pedrinho et al. 2024). Functional redundancy among organisms in these communities enhances the resilience of these communities to environmental stresses, allowing them to maintain essential processes like nitrification, denitrification, phosphorus mobilization, and pathogen suppression. However, the degree of diversity enhancement by organic management systems is also dependent on factors like soil type, climate region, and type of organic amendments used, which means that organic systems are not uniformly superior to conventional systems (Agyekum et al. 2023). Intensive conventional management systems, especially those that rely heavily on mineral fertilizers and pesticides, can also cause significant changes in the composition and function of rhizosphere microbial communities. These management systems can alter the structure of microbial networks, inhibit symbiotic colonization of plants, and reduce the abundance of functional genes involved in processes like nitrogen fixation and phosphorus mobilization (Oliveira et al. 2024).

Several important microbial processes play an essential role in the sustainable production of food from soybeans. Effective symbiotic colonization of soybean roots by *Bradyrhizobium* spp. is essential for biological nitrogen fixation, enabling the plant to meet its nitrogen requirements for optimal growth and development. AMF species, especially those of the genus Glomus, also have beneficial effects on soybean production. These fungi form an extensive network of branched hyphae that extend beyond the zone of depletion around the plant's root system, allowing it to access phosphorus and other minerals from the surrounding soil. The colonization of soybean roots by AMF also enhances the plant's tolerance to drought conditions (Peng et al. 2024). Other beneficial fungi, such as Trichoderma species, can act as biocontrol agents to prevent the colonization of soybean roots by harmful soil‐borne pathogens. They achieve this by using mechanisms such as antibiosis, mycoparasitism, and induction of systemic resistance (Jung et al. 2024). Other important microorganisms in the soybean rhizosphere include Actinobacteria, which include species of the genus *Streptomyces*. These bacteria are involved in the decomposition of organic matter and the production of antibiotics, which enhances the effects of other mechanisms that inhibit the growth of pathogens. Plant growth‐promoting rhizobacteria (PGPR) play a critical role in advancing sustainable soybean production. Bacteria such as Bacillus and Pseudomonas have beneficial effects on plants by enhancing their tolerance to abiotic stresses like drought and salt stress. These beneficial effects are mediated through multiple mechanisms, including the synthesis of phytohormones, accumulation of osmoprotectants, and the development of protective biofilms (Sharma et al. 2024a; Wu et al. 2024). Another important aspect of organic systems is microbial resilience, defined as the capacity of microbial communities to resist, adapt to, and recover from environmental disturbances. Enhanced SOM, aggregate stability, and buffered pH create a habitat for rhizobial nodulation and AMF colonization in the face of environmental stress (Akter et al. 2024). The effects of organic amendments can vary widely depending on factors such as nutrient composition, degree of amendment maturity, and site‐specific conditions, highlighting the importance of a context‐specific management approach.

Thus, organic management does have effects on the structure and function of the soybean rhizosphere, with alterations in major soil properties and the development of specific microbial assemblages. The effects of these changes on nutrient cycling efficiency, symbiont performance, and plant resilience place the rhizosphere microbiome at the heart of sustainable soybean production. This review synthesizes recent scientific evidence to (1) investigate the consistent effects of organic management on diversity, composition, and functional potential of rhizosphere microbes, (2) identify the dominant drivers of these changes, and (3) determine which microbe–rhizosphere interactions may provide the greatest future benefit for developing resilient and improving resilient low‐input soybean production systems.

## 2 Microbial Diversity, Composition, and Functions in Organic Soybean Rhizospheres

### 2.1 Diversity Patterns

Organically managed soybean rhizospheres typically exhibit higher levels of alpha diversity (richness and evenness) and greater complexity in co‐occurrence networks than conventionally managed rhizospheres (Agyekum et al. 2023; Pedrinho et al. 2024). This is often associated with the enrichment of bacterial phyla such as Proteobacteria, Actinobacteria, and Acidobacteria, as well as increased fungal diversity AMF. These microbial taxa are disproportionately responsible for nutrient cycling, organic matter degradation, and the suppression of pathogens. However, diversity gains are environment‐specific; the degree to which diversity is enhanced can vary according to factors such as soil texture, climatic conditions, composition of amendments, and historical impact of crop rotations (Crystal‐Ornelas et al. 2021; Fadiji et al. 2023). Organic management systems appear to foster greater levels of functional redundancy, as organic soils contain a wider range of taxa carrying genes that encode similar functions (such as those involved in nitrogen cycling, e.g., *nifH*, *amoA*, and *nirK*). Functional redundancy is known to enhance ecosystem resilience by stabilizing process performance following disturbance. Network analyses indicate that organic management enhances microbiome connectivity and reduces the influence of opportunistic species that are often overrepresented in chemically treated ecosystems (Oliveira et al. 2024).

### 2.2 Community Composition, Functional Activity and Biogeochemical Processes

In addition to diversity parameters, analyses of community composition reveal that the microbiome in soybean rhizospheres that are cultivated using organic management practices differs in its structure and composition from those found in conventionally managed rhizospheres. Such differences are associated with an enrichment of functionally relevant symbiotic and plant growth‐promoting (PGP) microorganisms. Symbiotic relationships involving rhizobia (Bradyrhizobium spp.) remain essential for the biological nitrogen fixation (BNF) process, as the efficiency of nodule‐based nitrogen fixation in rhizobia is directly related to soil pH stability and the availability of plant carbon (Haskett et al. 2022). Organic amendments tend to increase the density of rhizobial populations and enhance nod gene expression while minimizing the need for mineral nitrogen fertilizers, resulting in higher relative BNF rates compared with other management systems (Barbieri et al. 2023). In the context of phosphorus cycling, organic management systems commonly foster the development of AMF that expand soil hyphal networks beyond root zone depletion zones in soils. AMF also enhance phosphorous uptake by plants under low‐input conditions while improving water use efficiency (Peng et al. 2024). Organic management systems tend to foster greater colonization of AMF hyphae in soils, likely as a result of reduced fungicide exposure and enhanced soil aeration or structural heterogeneity. Organic management systems generally foster a high level of diversity in the Actinobacteria population that is rich in species of the genus Streptomyces. These bacteria play an important role in degrading complex organic matter in the soil while producing secondary metabolites that inhibit the activity (Almeida et al. 2023) of plant‐pathogenic bacteria and fungi. Trichoderma spp. are also present in organic management soils, functioning as beneficial biocontrol agents that affect pathogen suppression through mechanisms such as antibiosis, competition with fungal pathogens, mycoparasitism, and the induction of systemic resistance (ISR) through signaling pathways that include the jasmonic acid (JA) and ethylene (ET) pathways (Jung et al. 2024). Other types of beneficial bacteria, such as plant growth‐promoting rhizobacteria (PGPR), that have an advantage in terms of their ability to promote plant growth in organic systems, are also found in organic soybean management systems. These bacteria include species of the genera Bacillus and Pseudomonas that inhibit pathogens and promote plant growth through a variety of overlapping mechanisms. These mechanisms include the production of phytohormones (e.g., indole‐3‐acetic acid [IAAIB]), ACC deaminase activity, which helps plants tolerate stress by modulating ethylene levels, siderophores that promote the uptake of iron, and the formation of biofilms that contribute to improving plant growth and yield (Sharma et al. 2024a; Wu et al. 2024). Collectively, these functional guilds of microorganisms form the biological basis for the development of an organically managed soybean rhizosphere.

Functional assays and molecular assays for metabolic activity have confirmed that the biochemical activities in organic management systems are higher than those in conventional systems. Enzyme activities that indicate carbon cycling and nitrogen cycling, such as β‐glucosidase, urease, and acid and alkaline phosphatases, are often higher in organic than in conventional management soils. These elevated activities result in increased rates of substrate degradation and nutrient cycling in the organic system (Uwituze et al. 2022). Higher levels of microbial biomass carbon (C) and nitrogen (N) also confirm that these systems are capable of supporting a large amount of microbial metabolic activity (Joergensen et al. 2024). CO₂ fluxes that are determined by measuring soil respiration are also often higher in organic‐managed soybean rhizospheres than in conventional systems. Respiration rates are integrated measures of microbial activity that encompass both carbon cycling and the nutrient cycling processes that are occurring in the rhizosphere. Respiration rates are, therefore, high in organic systems that have recently been amended with fresh organic products (Mátyás et al. 2018). Analyses of functional gene abundance indicate that soils enriched with organic matter exhibit significantly higher copy numbers of genes associated with critical processes, including nitrogen fixation (*nifH*) and phosphorus mobilization (*pho*) (Igiehon et al. 2024). However, the enhanced rates of microbial respiration that occur in organic systems do not appear to result in improved carbon sequestration rates or processes; instead, rates of decomposition are increased to match the high rates of respiration that occur after the addition of fresh organic matter. The long‐term carbon sequestration potential of these systems will be revealed by measurements that track the stability of organic matter over time. Other measures of nutrient cycling efficiency (such as rates of nitrogen fixation and phosphorus solubilization) are often higher in organic systems than they are in conventional systems (Zhang et al. 2025). However, the nutrient release patterns of some organic amendments may not align with the developmental nutrient requirements of soybean plants, potentially resulting in trade‐offs or reduced nutrient‐use efficiency. The impact of nutrient cycling functions that are enhanced by organic management will thus be better understood in the context of nutrient availability rather than yield alone (Fontaine et al. 2024).

### 2.3 Microbial Resilience and Stability

Microbial resilience, the ability of a community to withstand or recover from disturbance, is a defining feature of sustainable agroecosystems. Organic soybean soils usually have higher structural stability, greater SOM content, and improved aggregate formation, creating microsites that support and protect the microbial community from the effects of disturbances (Ajiboye et al. 2022). Soil pH buffering in a favorable pH range (approximately 6.0–7.0) enhances rhizobial nodulation effectiveness and AMF colonization (Kabir et al. 2023). The reduced impact of chemically based disturbances in organic farming systems also protects the structure of the microbial community and its symbiotic members. In PGPR‐treated plants, enhanced biofilm formation in response to drought or salt stress increases the effectiveness of osmoprotectant synthesis to make plants more resistant to environmental stresses (Sharma et al. 2024a). Resilience should not be interpreted as an absolute characteristic, however, because it varies as a function of amendment quality, soil fertility level, and environmental conditions.

In general, organically managed soybean rhizospheres have greater microbial diversity, a greater abundance of beneficial symbiotic organisms, greater levels of certain enzymes, and improved nutrient cycling capabilities compared with the majority of conventional farming systems. These characteristics of the microbiome enhance nitrogen fixation efficiency, phosphorus acquisition, disease resistance, and environmental stress responses. However, these effects tend to vary in magnitude between different agroecological contexts, indicating a need to develop site‐specific management strategies (Agunbiade and Babalola 2024). By combining measurements of diversity, functional gene abundance, enzymes, and selected physicochemical properties of the soil, it is clear that microbial changes resulting from the introduction of organic amendments are consistent with resource heterogeneity and decreased chemical disturbance in the rhizosphere rather than random events that support the central role of rhizosphere microbiomes in soybean farming systems (Table 1).

**TABLE 1:** Impacts of microbe–rhizosphere interactions in organic soybean farming systems.

Functional microbial diversity	Enhances nutrient cycling, functional redundancy, and disease suppression	Promotion of symbiotic taxa (*Bradyrhizobium*, AMF), PGPR inoculation, diversified rotations	Fasusi et al. (2023); Li et al. (2024)
Organic soil amendments	Increase SOM, microbial biomass, and enzyme activity; improve soil structure	Compost, manure, legume cover crops, biochar, humic substances	Akanmu et al. (2020); Fornes et al. (2024); Joergensen et al. (2024)
Soil health management	Stabilizes microbial networks and aggregate formation	Reduced tillage, crop rotation, residue retention, organic matter incorporation	Alori et al. (2024); Toth et al. (2024)
Rhizosphere‐induced disease suppression	Activation of induced systemic resistance (ISR) and antagonistic inhibition of pathogens	*Trichoderma* inoculation, promotion of antagonistic bacteria, microbial volatiles	Jung et al. (2024); Liu et al. (2024)
Optimization of nitrogen fixation	Enhances nodulation efficiency and BNF gene expression	Effective rhizobial inoculants; maintenance of soil pH 6.0–7.0	Haskett et al. (2022); Alori and Babalola (2018)
Climate adaptation and stress resilience	Improves drought and salinity tolerance via phytohormones and osmoprotection	*Bacillus* and *Pseudomonas* inoculation; biofilm‐forming consortia	Sharma et al. (2024a); Wu et al. (2024)
Emerging applications	Microbiome engineering and precision organic management	Tailored microbial consortia; biomarker‐based soil monitoring	Khan et al. (2023); Mishra et al. (2025)

## 3 Drivers of Microbial Shifts in Organic Soybean Systems

Microbial community structure and functional potential in soybean rhizospheres result from the interplay between management‐induced drivers of community composition that act upon the availability of substrates and physicochemical conditions, as well as disturbance regimes. In organic farming systems, the reorganization of microbial communities is mainly determined by (i) organic amendments, (ii) reduced use of chemical materials, (iii) modified soil properties, and (iv) diverse crop management practices. The drivers interact synergistically to affect microbial assembly processes, functional gene expression, and biogeochemical cycling (Alori and Babalola 2018). The observed shifts in microbial communities are better interpreted as predictable responses to changed resource availability and disturbance regimes rather than assuming inherent advantages of organic systems.

### 3.1 Organic Amendments as Substrate and Habitat Drivers

Organic amendments, including compost, manure, green manure crops, crop residues, and biochar, represent primary bottom‐up drivers of community restructuring through their impacts on resource availability and habitat structure. Organic amendments increase the content of SOM, change the quality and composition of the carbon pool (labile vs. recalcitrant fractions), and alter the stoichiometric composition of nutrients, leading to changes in the types of metabolites produced and the competitive interactions between different microorganisms (Fornes et al. 2024; Joergensen et al. 2024). Compost and manure are diverse mixtures of easily decomposable organic compounds and stabilized fractions. The labile fraction stimulates the growth of copiotrophic bacteria in high numbers, such as species from the Proteobacteria and Bacteroidetes phyla. In contrast, the recalcitrant fractions promote the growth of fungi and actinobacteria involved in the degradation of lignocellulosic material. Higher SOM levels lead to higher mass per volume values of microbial biomass and increase the levels of certain enzymes, such as β‐glucosidase, urease, and phosphatase enzymes involved in the cycling of C, N, and P substrates (Liu et al. 2023).

Biochar not only affects the amounts of certain carbon‐containing compounds in the soil, but also modifies the structural properties of the amended soil. Biochar's high surface area and porosity promote aggregate formation, while its stable carbon compounds support micro‐aggregates that protect microbial communities from erosion and water stress. In general, the application of biochar has been shown to increase the abundance of certain groups of microorganisms (e.g., nitrifying bacteria) and the abundance of AMF, while also increasing the aeration of amended soils and stabilizing pH values (Fornes et al. 2024). However, the specific effects of different types of biochar on given properties are strongly dependent on the type of feedstock used to produce the biochar, as well as its pyrolysis temperature and the initial properties of the soil being amended.

Legume cover crops enhance their effects through the addition of biologically fixed nitrogen and carbon compounds derived from root respiration that promote AMF colonization (Igiehon et al. 2024). The increased carbon input allows for higher levels of energy‐rich substrates that correlate with higher levels of expression of genes for nitrogen (*nifH*) and phosphorus as in phosphate‐solubilizing microorganisms.

### 3.2 Reduction of Chemical Inputs and Disturbance Pressure

The exclusion of synthetic (inorganic) fertilizers, pesticides, and herbicides in organic farming systems leads to changes in nutrient gradients, as well as in the physical and biological impacts of disturbances. The heavy use of inorganic fertilizers (e.g., ammonium nitrate, diammonium phosphate) can negatively impact biological processes involved in nutrient acquisition. For example, the application of high rates of inorganic fertilizers, especially nitrates, can inhibit the process of symbiotic nitrogen fixation by depleting the rhizobia of resources, which leads to reduced nodule mass and *nif* gene expression (Agyekum et al. 2023). In general, the application of fertilizers can also disturb the function of beneficial organisms involved in nutrient acquisition. In contrast, the use of phosphorus‐based fertilizers is less effective, as the resulting increases in available phosphorus levels can inhibit the colonization of plants by mycorrhizal fungi.

Chemicals and herbicides can affect microbial processes directly and indirectly, for example, by causing damage to non‐target microbial species, as well as altering the physical/chemical and substrate‐based processes that affect community composition. Chemical‐based disturbances are reduced under organic management systems. Organic amended soils contain a greater diversity of functional microorganisms that are able to efficiently perform nutrient acquisition‐related processes; for example, phosphate‐solubilizing bacteria and AMF are more likely to persist in resource‐poor environments. However, the effective utilization of these available resources in organic systems requires the proper use of practices to ensure that nutrients are released from organic amendments at the right time to meet plant demand in order to avoid limiting their growth (Akanmu et al. 2021).

The effects of reduced disturbance on microbial communities are associated with changes that lead to the development of more stable communities over time. Network analyses of co‐occurrence patterns that have been applied to different soils have shown that organic systems tend to develop more complex and module‐rich network structures compared to mineral systems, which have been associated with increased stability (Oliveira et al. 2024).

### 3.3 Soil Physicochemical Modifications

Soil management practices significantly impact community composition and functioning through their effects on factors like soil structure, water regimes, and pH regulation. Increased SOM enhances the stability of soil aggregates through the development of stronger intra‐aggregate and inter‐aggregate bonds that provide more favorable conditions for microorganisms to thrive. Aggregate stability allows for the preferential preservation of beneficial organisms and their products, which improves the resilience of the soil microbial community to erosion and changes in environmental conditions (Rieke et al. 2022). The presence of more stable aggregate structures also improves conditions for aerobic and anaerobic processes to occur together in the same field by preventing the excessive formation of aggregate structures that limit oxygen penetration into certain areas. The development of different aggregate fractions also enhances the ability of the soil to hold water and air. In general, SOM contents above ≈2% improve the structure of the amended soil in ways that facilitate the development of functional microbial communities.

Increased SOM levels also improve the efficiency of symbiotic processes through effects on pH buffering. As a general rule, rhizobia are best able to survive and thrive in slightly acidic environments (with pH values near 6.0–7.0), while plants prefer slightly acidic (pH ≈6.0) to neutral conditions. Organic amendments that produce acidic byproducts after they decompose can, therefore, reduce the survival rate of rhizobia (Haskett et al. 2022). The buffering capacity of composted manure and other organic amendments can reduce the downward movement of H+ ions in acidic soils that would otherwise decrease their pH values to levels that inhibit root growth or impair the functioning of rhizobia. The use of byproducts from composted manures can also act as a buffer that helps stabilize pH values at beneficial levels.

### 3.4 Cropping Practices Influence Soil Microbiome

Cropping system design is also a key driver of rhizosphere microbial assembly. Cropping system design is recognized as a major driver of rhizosphere microbial assembly because differences in the design of cropping systems result in differences in root exudates and residues that, in turn, support different microbial communities and inhibit different pathogens (Crystal‐Ornelas et al. 2021). Increased diversity in the design of cropping systems tends to increase microbial richness and reduce the dominance of pathogens, especially pathogenic fungi (Adedayo and Babalola 2023).

Many different cropping system design choices can promote beneficial soil microbial communities. Crop rotation promotes heterogeneity in root exudates and residues, which supports a wide range of microbes while inhibiting pathogens. Cover cropping supports active belowground carbon and microbial biomass processes even in fertile farming zones by adding organic matter to soils and enhancing the impacts of both microbial biomass and carbon turnover on fertility (Moretti et al. 2024). Reduced‐till or no‐till cropping enhances soil microbial communities by minimizing mechanical damage to roots and associated microbes, which preserves fungal hyphae as well as microbial aggregates. The resulting soil habitats support beneficial effects on symbiotic relationships (Byers et al. 2024).

These cropping system design choices interact to exert plant‐mediated selection pressures within the rhizosphere. Selection pressures favor specific microbial community structures that facilitate efficient nutrient acquisition and utilize soil resource availability (Kuzyakov and Razavi 2019). Plant‐mediated selection favors organisms that produce nutrients or provide other beneficial services at the expense of organisms that compete for resources or damage roots (Byers et al. 2024). Organic amendments increase dependence between plants and microorganisms for available nutrients, which tends to enhance beneficial symbiotic relationships relative to mineral‐fertilizer dependent systems. However, specific effects may also vary according to differences in amendment quality, as well as differences in climate zone, soil type, and past management practices (Ishfaq et al. 2023).

### 3.5 Field and Greenhouse Experiments Show Management Drivers Map to Functional Outcomes

Field and greenhouse‐based experiments demonstrate causal relationships between management drivers and resulting functional outcomes in specific ecosystems. For example, Bradyrhizobium inoculum applications under organic management practices tend to enhance the efficiency of nodule formation and nitrogen fixation relative to high‐mineral‐nitrogen management practices (Rashid et al. 2014). Increased colonization of AMF in soil also tends to enhance phosphorus use efficiency in crops. Therefore, compost‐amended soils tend to have higher AMF colonization than unamended soils (Peng et al. 2024). The beneficial effects of biochar and compost amendments on microbial community processes are also well established. Biochar amendments have been shown to enhance populations of Trichoderma without suppressing other beneficial organisms, which have been shown to reduce disease incidence through mechanisms such as induced systemic resistance (Galeano et al. 2024). These types of functional outcomes demonstrate that structure changes to the microbial community resulting from management practices are not random events but are caused by specific drivers under a common set of rules.

The structure changes to the microbial community that result from the implementation of organic management practices are the result of integrated bottom‐up (resource‐driven) and top‐down (disturbance‐mediated) processes. Organic amendments increase the diversity of carbon substrates available for microbial growth and processing; chemical amendments decrease the pressure to select for beneficial organisms; physical conditions (e.g., soil structure) tend to increase habitat stability; and different cropping systems tend to increase the diversity of ecological niches in soils (Byers et al. 2024). These management practices tend to increase beneficial diversities while decreasing the diversity of pathogens or other detrimental organisms in the microbial community. The resulting microbial diversities, functional gene diversities, and ecological processes tend to increase beneficial functions such as nitrogen fixation by Rhizobia or phosphorus mobilization from AMF. The processes also tend to increase crop defense against pathogens and diseases caused by detrimental organisms in the community. The drivers of these changes are also often measurable, although the resulting impacts may differ depending based on factors such as amendment quality, as well as differences in climate zone, soil type, or past management practices (Bai et al. 2018; Beillouin et al. 2022; Smith et al. 2016).

## 4 Promising Microbe–Rhizosphere Interactions for Sustainable Soybean Production

Sustainable organic soybean production depends on functionally coherent plant‐microbe interactions that enhance nutrient uptake, defend against pathogens, and abiotically tolerate adverse conditions. The mechanisms and molecular, physiological, and ecological parameters that govern such interactions are also provided as deployable biological strategies to reduce the reliance on synthetic chemicals. Examples of consistently confirmed beneficial plant‐microbe interactions include symbiotic relationships with *Bradyrhizobium*, AMF, *Trichoderma* species, and plant growth‐promoting rhizobacteria (PGPR), such as Bacillus and Pseudomonas species, as well as Actinobacteria (formerly known as Streptomyces) (Agbodjato and Babalola 2024; Peng et al. 2024).

The benefits of these beneficial microbes extend beyond simply promoting plant growth. They also reconfigure rhizosphere signaling networks, activate functional genes, and affect soil biogeochemical processes. This chapter summarizes four key plant–microbe interactions that occur in soybean systems. These include:

- Symbiotic nitrogen fixation with *Bradyrhizobium* spp.: The major organism responsible for fixing atmospheric N_2_ into forms usable by plants.
- Phosphorus acquisition and stress mitigation with AMF: These fungi are essential for phosphorus acquisition in low‐fertilizer organic systems.
- Biological control and induced systemic resistance with *Trichoderma* spp. These fungi act as antagonists against a variety of pathogens but also elicit systemic resistance in their hosts.
- Plant growth‐promoting bacteria (PGPR): Bacillus, Pseudomonas, and related bacteria enhance plant productivity by producing phytohormones, fixing iron compounds, and reducing stress.

Each of these sections summarizes the main characteristics of the organism involved, followed by an overview of the key benefits provided by these microorganisms to their soybean hosts, and the relevant mechanisms that underlie these effects. All sections conclude with implications for organic agriculture (Adedayo and Babalola 2023; Akanmu et al. 2025).

### 4.1 Symbiotic Nitrogen Fixation: Bradyrhizobium–Soybean Interaction

The symbiotic relationship between Bradyrhizobium spp. and soybean ( *Glycine max* ) is the most critical biological source of nitrogen for organic systems based on legumes. The presence of flavonoids in bean roots initiates a signaling cascade that leads to the colonization of root hairs by Bradyrhizobium and the induction of nodule formation. In these nodules, the bacteria reduce atmospheric N_2_ into ammonia (NH_3_) with nitrogenases encoded by *nif* and *fix* genes. The ammonia is used by the bean plant to synthesize amino acids, thus providing a non‐synthetic source of nitrogen that would otherwise require expensive and environmentally damaging methods to produce.

Organic nutrient production systems tend to enhance the efficiency of symbiotic nitrogen fixation (SNF) compared to systems that rely on inorganic fertilizers. For example, high levels of mineral nitrogen can suppress nodule development by creating feedback effects (Zhao et al. 2022). Compost‐rich systems tend to promote *nifH* gene expression in root nodules and increase the stability of these symbiotic relationships. Organic systems also tend to develop soil physical properties and pH buffering capacities that favor rhizobia populations. Soybean yields in organic systems, therefore, primarily depend on effective inoculation with Brady rhizobia strains that are compatible with the selected bean genotype, as well as adequate carbon supply to nodules for energy production.

### 4.2 Phosphorus Acquisition and Stress Mitigation: Arbuscular Mycorrhizal Fungi

Arbuscular mycorrhizal fungi (AMF), especially those belonging to the genus Glomus, form symbiotic relationships with plants that result in the formation of arbuscules in the infected plant roots. These arbuscules develop from branching hyphae that penetrate cortical cells and ultimately lead to the development of a network of mycorrhizal fungi in the plant root system that eventually forms a symbiotic relationship with the bean plant. The fungi provide phosphorus and trace minerals like zinc to the bean plant in return for carbohydrate products.

The acquisition of phosphorus is a particularly important mechanism in low‐fertilizer organic systems because the effective use of sparingly soluble inorganic phosphorus compounds is limited. AMF can absorb these sparingly soluble phosphorus compounds from their soil microenvironments. Their hyphae can extend many kilometers into low‐phosphorus areas beyond the depletion zone that surrounds bean roots. Organic systems also increase the size of the effective surface area that bean roots have for absorbing phosphorus compounds. The presence of organic matter in these systems also increases the rates at which organic acids and other organic compounds can dissolve bound phosphorus compounds in soil particles. Studies have shown that AMF can increase the yield of bean plants under organic management systems (Peng et al. 2024; Adedayo and Babalola 2023). Organic systems enhance AMF colonization by increasing the soil's effective surface area for phosphorus acquisition and improving the efficiency with which AMF can utilize sparingly soluble phosphorus sources. AMF colonization also improves water use efficiency in bean plants by modulating plant gene expression that controls phosphate transporter activity and osmotic balance in the plant's cells. The colonization of roots by AMF is influenced by factors like soil phosphorus concentrations, host genotype compatibility, and tillage practices that influence soil structure and organic matter content. Organic farming systems, therefore, need to implement practices that promote AMF in their bean crops over multiple growing seasons (Peng et al. 2024).

### 4.3 Biological Control and Induced Systemic Resistance: Trichoderma spp.

Trichoderma species are beneficial fungi that act as biological antagonists to a wide variety of pathogenic fungi in soil and also elicit induced systemic resistance (ISR) in their hosts. The species produce a wide variety of enzymes, secondary metabolites, and antibiotics that kill or inhibit the growth of pathogenic fungi in the soil. They also trigger ISR effects in bean plants through signaling pathways that involve ethylene and jasmonic acid. These signaling pathways lead to the expression of defense genes in bean plants without causing negative effects on bean productivity. The early colonization of bean roots by Trichoderma species not only increases resistance to pathogens but also improves root development, enhances root system efficiency, and improves nutrient uptake efficiency by improving root architecture. Organic systems tend to foster a high population of Trichoderma species in the rhizosphere because they do not contain fungicides, which kill beneficial fungi, and their soil conditions foster the development of organic matter that Trichoderma species can use as a source of nutrients to support their growth (Jung et al. 2024; Galeano et al. 2024). Organic systems, therefore, enhance the potential benefits of using Trichoderma species as biological control agents by increasing their populations in the rhizosphere. Trichoderma species can be implemented as microorganisms that can be introduced into organic farming systems to act as beneficial agents that can enhance yields without causing adverse effects on the environment or the ecosystem.

### 4.4 Plant Growth‐Promoting Rhizobacteria: Bacillus and Pseudomonas

Plant growth‐promoting rhizobacteria (PGPR) are beneficial bacteria that can increase yields and improve plant productivity. These bacteria belong to several different genera, but the most important genera with respect to bean productivity are Bacillus and Pseudomonas. The bacteria increase plant productivity by acting as phytohormone producers that release plant hormones such as indole‐3‐acetic acid, which stimulates plant growth and development. The bacteria can also fix iron compounds, thus increasing yields when soil iron concentrations are limited. The bacteria also produce enzymes that break down plant metabolic byproducts, such as acetate, which limits plant growth processes. They can also produce enzymes that break down polyphenols, thus increasing the availability of nutrients and other resources for plant growth. The bacteria can also use a deaminase enzyme to break down ACC (1‐aminocyclopropane‐1‐carboxylate), which is a byproduct of plant metabolism that limits plant growth processes. This deaminase enzyme breaks down ACC into ammonia and alpha‐ketobutyrate, thus reducing its adverse effects on plant growth processes. The deaminase enzyme increases the availability of nutrients and other resources that are required for plant growth processes (Akanmu et al. 2025).

The inoculation of bean plants with stress‐adapted PGPR strains can improve yield stability in challenging environmental conditions, such as drought conditions or high salinity conditions. For example, studies have shown that yield stability improves in conditions where the relative humidity of the air is high because the bacteria improve stomatal density and other yield‐related traits (Sharma et al. 2024a; Wu et al. 2024). The bacteria can also improve yield stability by improving photosynthetic efficiency and enhancing chlorophyll content and other physiological processes. The bacteria also improve yield stability by enhancing nutrient uptake efficiency, thus providing more nutrients and resources that can be used to improve growth processes and yield traits. The benefits that PGPR bacteria provide to bean plants are achieved through their metabolic effects on their hosts, thus allowing them to act as beneficial agents in organic farming systems that can increase yields without causing adverse effects on ecosystems or environments.

### 4.5 Actinobacteria and Functional Redundancy: Streptomyces

Actinobacteria (Streptomyces) are also involved in organic matter degradation and pathogen control. Filamentous bacteria such as Streptomyces produce exoenzymes that break down complex polymers like cellulose and chitin, and many strains produce antibiotics and antifungal compounds that inhibit pathogens. Organic soybean rhizospheres tend to have higher densities of Actinobacteria, for example, and often display enhanced nutrient mineralization rates and decreased disease incidence (Wu et al. 2024). Their combined functions of nutrient cycling and biocontrol enhance the functional redundancy of the microbial community, improving the community's resilience to environmental disturbances.

### 4.6 Integrated Functional Outcomes

The combination of biological nitrogen fixation, mycorrhizal fungi‐mediated phosphorus acquisition, ISR‐mediated disease prevention, and PGPR‐mediated stress adaptation (and even the, as yet, less integrated role of Actinobacteria in nutrient recycling) suggests a set of functionally robust pathways for sustainable intensification of soybean production. The effectiveness of those roles, however, is contingent on ecological compatibility, soil physicochemical conditions, and host genotype (Akanmu et al. 2021). Future optimization of such inoculation or amendment systems should therefore focus on designing inocula that incorporate both conserved and novel microorganisms, rather than viewing introduced microorganisms as alien entities competing with existing microbial populations. The most promising long‐term approach may therefore be to use multi‐strain consortia that exploit all possible symbiotic effects between different types of symbionts (Akanmu et al. 2025).

## 5 Soil Health Indicators Associated with Microbial Dynamics

Soil health in soybean production systems is inextricably linked with microorganisms. A set of quantifiable biological and physicochemical parameters can be used to evaluate and compare organic management effects on rhizosphere functioning. Parameters include microbial biomass, diversity metrics, enzyme activity, respiration, nutrient cycling genes, SOM, pH stability, and functional aggregate stability. These parameters relate to both the structural and functional aspects of the soil ecosystem.

### 5.1 Microbial Biomass and Community Structure

Microbial biomass carbon (MBC) and nitrogen (MBN) are indicators of living organic matter and serve as early indicators of changes in biomass due to management. Organic amendments increase the availability of labile carbon that supports microbial growth. Phospholipid fatty acid (PLFA) profiling and quantitative PCR (qPCR) analyses show high levels of biomass and a wide variety of functional guilds in organic soybean systems (Joergensen et al. 2024). Higher levels of biomass are associated with increased decomposition capacity and nutrient cycling efficiency. However, biomass levels are not sufficient to serve as indicators of functional efficiency; therefore, diversity and enzyme activity are also important.

### 5.2 Diversity and Redundancy of Functional Microbes

High‐throughput sequencing has shown that organic systems usually have a higher level of alpha diversity and more complex co‐occurrence networks than conventional systems (Pedrinho et al. 2024). High levels of Proteobacteria, Actinobacteria, and Acidobacteria are associated with increased diversity and redundancy of functional capabilities. Higher diversity in nutrient cycling processes helps maintain system stability. Diversity metrics, in contrast to other parameters, are less susceptible to over‐explaining the effects of intensive chemical management that can simplify ecological networks.

### 5.3 Soil Enzymes as Functional Indicators

Soil enzymes are key indicators of the functional capabilities of microorganisms. β‐glucosidase activities relate to carbon cycling processes, urease activity relates to nitrogen cycling, and phosphatase activity is associated with phosphorus availability. Organic amendments increase enzyme activities by providing substrates for microbial growth (Liu et al. 2021). Enzyme assays are sensitive and rapid indicators of changes in management practices, often more responsive than bulk chemical composition measurements.

### 5.4 Soil Respiration Rates

Soil respiration rates are indicators of the metabolic activity of microorganisms. Basal and substrate‐induced respiration rates measure the intensity of respiration through CO_2_ release. Organic soybean systems typically have higher respiration rates than conventional systems, indicating increased rates of carbon turnover (Mátyás et al. 2018). Increased rates of respiration are associated with increased levels of biological activity. However, excessively high rates may indicate unstable carbon cycling processes that result in excessive loss of carbon. Respiration rates should therefore be evaluated relative to the input levels of carbon and carbon use efficiency.

### 5.5 Nutrient Cycling Processes

Microorganisms play a key role in most nutrient cycling processes, such as nitrogen fixation, nitrification, denitrification, and phosphorus solubilization. Organic management enhances the efficiency of biologically mediated nutrient cycling processes. Organic amendments increase the abundance of functional genes that encode enzymes involved in processes such as nitrogen fixation (*nifH*), nitrification (*amoA*), denitrification (*nirK*), and phosphorus solubilization (*phoD*) (Igiehon et al. 2024). These gene abundance measures provide insights into the efficiency of different nutrient cycling processes beyond the information provided by conventional chemical tests for soil health.

### 5.6 Physical Structure and SOM

Soil organic matter (SOM) is an important indicator of the stabilizing structure of the soil. Organic amendments increase SOM levels through the accumulation of plant residues and microbial biomass. Increased SOM levels are associated with increased levels of microbial biomass, improved aggregate stability, and enhanced functional properties, such as water retention capacity (Ajiboye et al. 2022). Loss‐on‐ignition (LOI) and wet oxidation analyses confirm that organic soybean systems have higher SOM levels than conventional systems.

Aggregate stability is enhanced due to the presence of fungal hyphae and microbial exopolysaccharides. Stable aggregates with high pore volumes protect the soil ecosystem against erosion while maintaining habitats for beneficial microorganisms (Moretti et al. 2024). Stable aggregates improve the infiltration of water into the soil while reducing the leaching of nutrients. Improved physical properties contribute to sustainable system performance.

### 5.7 Soil pH Stability

Soil pH stability is essential for developing optimal nutrient availability patterns and for maintaining the homeostasis of the microbial community. Organic amendments tend to buffer acidic or basic soil conditions to levels that are optimal for soybean growth (approximately pH 6.0–7.0) (Haskett et al. 2022; Kabir et al. 2023). Stable pH values improve the efficiency of nutrient availability and utilization processes while creating optimal conditions for symbiotic interactions between soybean roots and rhizobia.

The various indicators of effective functioning of the rhizosphere in organic soybean systems include (1) microbial biomass, (2) diversity metrics, (3) enzyme activity, (4) respiration rates, (5) gene abundance for nutrient cycling enzymes, (6) SOM levels, (7) pH stability, and (8) functional aggregate stability. The different indicators relate to distinct aspects of the functioning of the system. Microbial biomass and diversity metrics relate to the structural aspect of the ecosystem, whereas enzyme activity and respiration rates relate to functional aspects. The different indicators can be evaluated using established sampling and analysis methodologies. However, the interpretation of results requires careful consideration of the context in which the soybean systems being compared have been established. Outcomes should therefore be evaluated relative to factors such as climate conditions, soil types, and management intensities (Akanmu et al. 2021).

## 6 Comparative Synthesis: Organic and Conventional Soybean Rhizosphere Systems

The meta‐analysis compares conventional and organic soybean production systems regarding their management‐dependent differences in the assembly of microbial communities, functional genes, and soil health parameters. It proves more scientifically sound to interpret results as context‐specific responses to the type of nutrient input, disturbance, and carbon management rather than to view organic compared to conventional systems as an inherently superior or inferior production paradigm. This meta‐analysis combines information on the structure and composition of microbial communities and their functions, as well as plant–microbe interactions, to extract consistent patterns and their causal relationships.

### 6.1 Microbial Diversity and Community Composition

Organic compared to conventional soybean rhizosphere soils exhibit significantly higher levels of alpha diversity and more complex co‐occurrence networks. Organic amendments (e.g., compost) increase the abundance of Proteobacteria, Actinobacteria, and Acidobacteria populations, which are associated with nutrient cycling and decomposition and stress tolerance (Agyekum et al. 2023; Pedrinho et al. 2024). Higher evenness of microbial populations increases the number of functional redundancies that decrease the vulnerability of the microbiome to disturbances.

Comparatively, the use of intensive mineral fertilizers and pesticides in conventional production systems tends to simplify the microbiome and promote the growth of fast‐growing, high‐flux copiotrophic or stress‐tolerant bacteria (Byers et al. 2024). However, diversity estimates can vary according to fertilizer application rates, tillage practices, and other management practices. Reduced‐input conventional systems may exhibit intermediate diversity levels, emphasizing the need to differentiate between high‐input and conservation‐oriented conventional practices.

### 6.2 Functional Potential and Enzymatic Activity

Functional parameters are more informative than taxonomic classifications. Organic soybean production systems exhibit higher levels of activity of β‐glucosidase, urease, and phosphatase enzymes involved in carbon degradation and nutrient cycling than conventional production systems (Liu et al. 2021). Organic amendments increase the size of the microbial biomass and the amount of nutrients made available to the growing plants.

Organic soils relative to conventional soils exhibit higher rates of basal respiration, which measures microbial activity (Mátyás et al. 2018). However, the relevance of these observations is contingent on carbon use efficiency; increased respiration does not directly reflect greater carbon sequestration potential.

In conventional systems based on synthetic fertilizers, nutrients are provided exogenously; hence, expression of functional genes related to processes such as biological nitrogen fixation (*nifH*) and phosphorus solubilization is decreased in environments with high levels of available nutrients (Igiehon et al. 2024). However, modern approaches to integrated nutrient management can limit the effects of these changes.

### 6.3 Nutrient Cycling Dynamics

Nitrogen cycling represents one of the most distinct processes differentiating organic and conventional production systems. Organic systems predominantly depend on symbiotic nitrogen fixation mediated by *Bradyrhizobium* spp., whereas conventional systems largely rely on mineral nitrogen inputs and the decomposition of organic matter to supply plant‐available nitrogen forms. Consequently, organic management practices tend to foster more diverse and functionally complex rhizosphere microbiomes in soybean systems, while conventional systems generally support comparatively less diverse microbial communities (Akanmu et al. 2021).

Phosphorus cycling contrasts between organic and conventional systems. Organic systems use AMF as an important agent for phosphorus recovery in low‐input systems. Conventional systems use soluble phosphorus fertilizers, which limit the role of AMF in plants by limiting the allocation of plant carbon to the fungi; thus, colonization of the plants by AMF is reduced (Haskett et al. 2022; Peng et al. 2024). These changes indicate that the form of nutrients is an important factor in the changes observed in microbial interactions with plants.

### 6.4 Soil Structural and Biophysical Indicators

Soil organic matter (SOM) content and aggregate stability are two structural indicators that consistently show differences between organic and conventional production systems. Long‐term organic farming systems increase SOM through organic amendments such as compost; the resulting increases in microbial biomass support better SOM persistence and soil structural stability (Joergensen et al. 2024). Increased levels of microbial products increase SOM persistence. Soil microorganisms produce exopolysaccharides, while fungi produce long hyphae that help to bind aggregates. The stability of these aggregates increases with soil water potential; thus, improved structure enhances infiltration and reduces erosion potential (Li et al. 2025).

Improved SOM persistence in organic compared to conventional farming systems is because SOM is less susceptible to degradative enzymes; thus, SOM persistence can increase with increasing SOM levels (Mao et al. 2022). Improved SOM persistence improves structure (Hallett et al. 2022). In contrast, intensive tillage practices that exhaust soil resources and limit residue retention in some conventional farming systems increase SOM degradation and reduce SOM levels (Bezboruah et al. 2024). Tillage increases the degradation rates of organic matter in the toposit. Thus, it should be emphasized that the type of conventional farming practice is more important than its label. Conservation tillage with residue retention can partially counteract the detrimental effects of degrading SOM; thus, it improves the beneficial structural effects of SOM (Sarker et al. 2022).

### 6.5 Plant–Microbe Symbioses and Stress Resilience

Symbiotic interactions between plants and their associated microorganisms differ between organic and conventional farming systems (Akanmu et al. 2025). Organic farming systems increase the size of the soil microbiome, which improves nutrient availability for plants and improves their health and yield (Galeano et al. 2024; Peng et al. 2024). Improved availability of nutrients also supports enhanced resistance of plants to environmental stresses. Improved root health increases the size of root‐associated microbial communities (Galeano et al. 2024), while improved root exudate quality supports beneficial interactions with rhizosphere microbes (Haskett et al. 2022). These changes support better nutrient use efficiency, better tolerance of abiotic stress factors, and better yield potential.

The increased abundance of beneficial fungi, especially *Trichoderma*, also supports the development of plant‐induced systemic resistance (ISR) in organic systems (Haskett et al. 2022; Peng et al. 2024). Reduced chemical disturbance increases the size of populations of mutualistic microbes that persist in the soil. Better symbiosis in organic farming systems is because organic amendments favor the development of beneficial microorganisms, whereas in conventional farming systems, frequent use of chemical pesticides and fertilizers disrupts beneficial interactions between plants and their microbiomes (Migliore et al. 2022). Conventional systems can reduce the intensity of symbiotic relationships when high levels of fertilizers and pesticides kill off the microbes. However, inoculation and reduced‐input strategies can restore the functional relationships. In the case of abiotic stress, organically‐managed soils with higher SOM and a greater diversity of PGPR (such as Bacillus and Pseudomonas) tend to exhibit enhanced drought tolerance and antioxidant defense responses (Sharma et al. 2024a; Wu et al. 2024). Increased water retention and the formation of microbial biofilms enhance the resistance of the system to stresses. Yet, yield outcomes under extreme conditions remain largely determined by the choice of cultivar and region (Table 2).

**TABLE 2:** Comparative overview of microbial dynamics in organic and conventional soybean rhizospheres.

Microbial diversity	Often higher richness and network complexity; enrichment of functional taxa including *Bradyrhizobium*, AMF, and Actinobacteria	Diversity may decline under high chemical input; simplified networks under intensive management	Agyekum et al. (2023); Pedrinho et al. (2024)
Community composition	Greater representation of symbiotic and decomposer guilds	Increased dominance of copiotrophic or stress‐tolerant taxa in high‐input systems	Byers et al. (2024)
Enzymatic activity	Elevated β‐glucosidase, urease, phosphatase; active organic matter turnover	Lower activity where nutrients supplied exogenously; variable under conservation practices	Liu et al. (2021)
Nitrogen cycling	Strong biological N fixation; reliance on microbial mineralization	Mineral N fertilization reduces dependence on fixation	Igiehon et al. (2024)
Phosphorus dynamics	Enhanced AMF‐mediated acquisition under low‐input conditions	Soluble P fertilizers may reduce mycorrhizal colonization	Haskett et al. (2022); Peng et al. (2024)
Soil organic matter	Generally higher SOM and improved aggregate stability	SOM may decline under intensive tillage; conservation practices mitigate losses	Joergensen et al. (2024)
Stress resilience	Greater microbial buffering capacity under drought/salinity	Resilience varies; often dependent on external inputs rather than biological buffering	Bisht et al. (2025)

**FIGURE 1:**
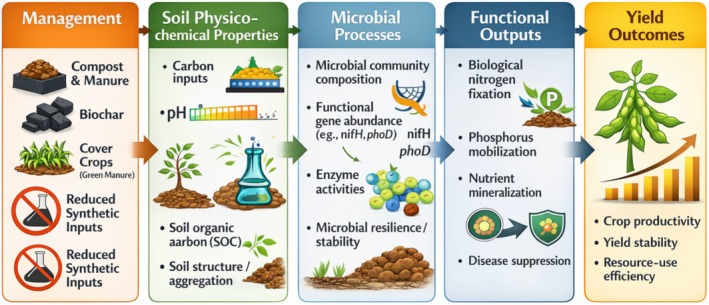
Mechanistic synthesis linking management practices to yield outcomes through soil physicochemical regulation and microbial gene expression.

The comparative evidence indicates that organic soybean production systems promote biologically mediated nutrient cycling, greater microbial diversity, and enhanced structural stability. The mechanisms underlying these effects relate to carbon, disturbance, and the strength of the symbiotic relationships established. Conventional production systems, particularly those that follow a high‐input strategy, are more likely to reduce the dependence of the microbial community on each other by substituting synthetic products for many of the services that the microorganisms would otherwise perform. Conservation‐oriented conventional farming can reduce the gap that opens up by following this strategy. Thus, instead of seeing a categorical distinction between the two approaches, it is possible to identify a set of management principles that are more likely to yield functionally integrated rhizosphere communities. Long‐term sustainability of the productivity of these systems is therefore less a question of system label and more a question of which management strategies pay more attention to the maintenance of the processes by which the microbial community mediates the functioning of the system. Figure 1 illustrates the mechanistic pathway from management to yield through soil physicochemical processes and microbial gene expression. The figure illustrates how management practices such as the use of organic amendments, the avoidance of certain chemical products, and conservation strategies affect soil properties like the concentration of carbon, pH, element stoichiometry, and aggregate stability. In turn, the physical and chemical conditions of the soil determine which microbial functional genes are expressed (e.g., *nifH*, *phoD*, and many other genes involved in nutrient cycling) (Akanmu et al. 2025). The functions enabled by those genes, such as biological nitrogen fixation or phosphorus release, lead to increased yield stability, resource‐use efficiency, and long‐term sustainability of soybean production systems.

## 7 Future Directions and Applications

The mechanistic evidence synthesized in this review demonstrates that management‐induced changes to rhizosphere microbial communities can drastically alter nutrient cycling, stress adaptation, and soil physical stability in soybean production systems. The important next step is to translate these ecological insights into practical, scalable, reproducible, and economically viable interventions. Future research and application should focus on microbiome engineering, functional inoculant design, biomarker‐enabled soil health diagnostics, climate‐resilient microbial strain deployment, and integration with precision agriculture technologies.

### 7.1 Microbiome Engineering and Synthetic Consortia

Microbiome engineering seeks to actively influence soil assembly processes to enhance the functional performance of the microbiome. This approach seeks to identify main species that coexist with soybean crops and design appropriate microbial consortia to ensure their establishment in the rhizosphere. Potential target organisms for engineering include Bradyrhizobium (N‐fixing), AMF (e.g., Glomus spp.; phosphorus acquisition), Trichoderma (biocontrol and induced systemic resistance), and Actinobacteria (e.g., Streptomyces; nutrient recycling, pathogen suppression).

Recent studies demonstrate that multi‐strain microbial consortia provide superior performance to single strains, enabling the exploitation of functional redundancy and ecological synergy (Khan et al. 2023; Galeano et al. 2024). For example, the application of nitrogen‐fixing bacteria, phosphorus‐solubilizing microorganisms, and fungi capable of inducing systemic resistance can simultaneously enhance soybean nodulation and nutrient acquisition while protecting against disease. Yet applicability studies must consider factors such as strain compatibility, colonization dynamics, competition exclusion, and persistence when transferring these findings to field conditions (Akanmu et al. 2021). Long‐term field‐scale experiments and studies of genotype‐specific matches are needed for future research.

### 7.2 Tailored Organic Amendments and Biostimulants

Organic amendments are evolving beyond their original function as general‐purpose fertilizers or conditioners. Current trends in their production and processing now enable the development of tailored amendments for targeted effects. Biochars and composts, two of the most promising amendment types, can be engineered to exhibit desired properties. For instance, the reductive capacities of certain byproducts or waste materials can be harnessed to create a specific range of redox potential values. The resulting biochars then provide optimal habitats for AMF (Fornes et al. 2024; Uzoh et al. 2025). Compost products, on the other hand, can be designed based on specific requirements. For example, composts derived from legume residues or enriched with specific microbial strains can be formulated to actively promote the growth of N‐fixing microorganisms or to release phosphorus (P) ions.

Another promising interface between microbiology and agronomy is biostimulant technology. Biostimulants can be defined as processed organic products or active microorganisms that stimulate an organism's natural processes (Akanmu et al. 2023). Metabolites, humic substances, and plant extracts are increasingly recognized as inexpensive sources of valuable nutrients for microorganisms. The impact of these amendments on the composition and function of microbiomes in agricultural ecosystems has only begun to be elucidated (Peng et al. 2024). The integration of these amendments into a broader framework involving the deliberate introduction of specific microbial species can develop new strategies for improving root system development and enhancing soil microbiome size.

### 7.3 Microbial Biomarkers and Decision‐Support Diagnostics

The emergence of data‐driven approaches to managing soil health poses essential questions about how to identify and quantify the biological parameters critical to productivity. Reliable biological indicators or “Biomarkers” are essential for successfully transitioning to data‐driven soil management systems. Biomarkers are quantifiable yet informative parameters that can be measured cost‐effectively and practically. Enzyme activities such as β‐glucosidase and urease can serve as important indicators of the potential of the present microbial populations to perform beneficial functions (Ciuffreda et al. 2024). In situ estimates of microbial biomass carbon (C) and analysis of phospholipid fatty acid (PLFA) patterns can also provide useful insights into the presence of specific microbial groups. Functional genes such as *amoA* (nitrogen fixation), *nifH* (ammonium assimilation), and *phoD* (phosphorus utilization) are also becoming increasingly recognized as reliable biomarkers for managing soil fertility or assessing microbiome health.

Recent developments in fast sequencing technologies have made it possible to incorporate such new biomarkers into routine soil health tests. The implementation of a set of well‐defined parameters that define threshold values will enable producers to monitor changes in their biological parameters alongside other essential performance metrics in a user‐friendly format. The integration of decision support tools into monitoring and management protocols will help producers predict the desired adjustments to their management practices (e.g., adjusting rates of specific amendments or inocula).

### 7.4 Climate‐Resilient Microbial Deployment

The effects of climate change are altering the types of biotic stresses that agricultural microbiomes must contend with. In addition to long‐term effects such as atmospheric temperature rise, ocean acidification, and altered global weather patterns, climate change is becoming increasingly associated with short‐term extreme weather events like droughts, floods, and storms (Agunbiade and Babalola 2024). The development of microbiologically enhanced crop resilience to abiotic stresses represents a promising route forward for coping with these changes. Plant growth‐promoting rhizobacteria (PGPR) have already demonstrated their effectiveness in improving osmotic tolerance, upregulating antioxidant defenses, and optimizing root development. Studies have shown that Bacillus and Pseudomonas strains can enhance their host plants' ability to withstand osmotic stress by inhibiting oxidative processes and improving nutrient uptake (Bisht et al. 2025). Another relevant example is the use of Trichoderma species, which have repeatedly proven their value as biocontrol agents for improving plant resilience to abiotic stresses. These fungi can positively interact with their hosts by stimulating beneficial physiological changes and supporting the development of improved root systems and enhanced nutrient efficiency. In addition, recent studies have demonstrated that Trichoderma species can enhance osmotic tolerance to salt stress and improve nutrient absorption efficiency in salt‐damaged tissues (Sharma et al. 2024b).

In identifying suitable strains for the development of stress‐adapted functional microbes, however, researchers must consider additional factors. The selection process requires careful consideration of factors such as the potential tolerance of target organisms to environmental stresses and the presence of pathogens that could limit the effectiveness of introduced species (climate‐resilient PGPR). The potential long‐term effects of introducing specific functional microorganisms or constructing synthetic consortia on the stability of native microbial populations are another essential consideration. To achieve optimal effectiveness while minimizing potential risks, the development of stress‐adapted functional microbes should be combined with other approaches capable of improving the resilience of crops to adverse environmental conditions. The integration of functional microbes that actively mitigate negative effects on plant productivity with breeding efforts focused on the development of more resilient crop species could lead to notable improvements in yield stability.

### 7.5 Integration With Precision and Digital Agriculture

The integration of microbiology into precision agriculture is an emerging area of research and development that seeks to combine insights and methods from a range of different fields to achieve better outcomes for food production. Remote sensing technologies and advanced geospatial modeling approaches can be combined with high‐throughput DNA sequencing techniques and advanced statistical models and machine learning algorithms to create a new generation of tools for predicting microbial performance in a variety of different agricultural ecosystems (Mishra et al. 2025). These spatially explicit models can be designed to predict the locations of key microorganisms in the rhizosphere throughout the entire growth cycle of a crop. The information generated by such models can be integrated into practical advisory systems that provide actionable guidance to producers on where to apply specific microbial amendments or other interventions. For instance, models that accurately predict the presence of beneficial microbial organisms in the rhizosphere will enable producers to target their applications for the development of improved soil microbiomes.

A more practical strategy is to incorporate strategies for optimizing the use of organic amendments into existing precision agriculture systems, which can be integrated with modern geo‐statistical models to guide their optimal placement. Prioritization of the application of amendments to the agricultural areas with the highest levels of microbiome stress and lower levels of microbial biomass will be more efficient for achieving long‐term sustainability goals than the use of standard, blanket strategies for applying organic amendments (Agunbiade and Babalola 2024). This framework will help producers avoid inefficient practices and high costs. The use of advanced technologies for data analysis may also provide new insights into the ways in which various functional groups of microorganisms interact within the rhizosphere and affect the productivity of crops. The identification of the essential characteristics of the various types of habitats that develop in the rhizosphere will enable the development of strategies for creating optimal conditions for the different types of microorganisms that play beneficial roles in food production. The integration of organic amendments and specific functional microbial species into the framework of precision agriculture and other digitalized approaches to food production will lead to the development of new concepts for precision organic management (POM) that can be adjusted to accommodate all types of agricultural systems and production practices.

### 7.6 Strategic Focus for the Future

The strategic focus for the future is around three intersecting but prioritized areas of work that need to be maintained together:

- Functionally designed and validated multi‐strain consortia microbiomes.
- Biomarker‐based management of microbiomes in real time, linking microbial health to operational decisions.
- Climate‐smart use of stress‐tolerant microbes in combination with precision agriculture technologies.

All of this will require a multidisciplinary team of experts in the fields of soil microbiology, plant physiology, bioinformatics, and agronomy. The focus for the future needs to shift from a bottom‐up, descriptive community ecology approach to one that is predictive and functionally oriented, linking microbial gene expression to measurable impacts on yield and resilience.

## 8 Conclusion

Organic management systems affect the diversity, composition, and function of the rhizosphere microbiome of soybean. Organic amendments such as compost, manure, and biochar convert into SOM while also stabilizing aggregate structure, buffering pH, increasing microbial biomass, increasing substrate enzyme activity, and reorganizing the microbial community to enhance the availability of nutrients, nitrogen fixation, phosphorus solubilization, and disease suppression. The drivers of the reorganization of rhizosphere microbiomes in organic soybean farms have now been elucidated. Bottom‐up resource input effects from organic amendments, minimal chemical disruption, and changes in soil physicochemical properties, along with increased crop diversity, act in concert to restructure the rhizosphere microbiome. These factors guide functional gene expression, influence microbial network structure and biogeochemical cycling, and reveal predictable and anticipated rather than random outcomes.

A variety of interactions in the soybean microbiome may hold the greatest promise for long‐term sustainability of food production systems. Symbiotic relationships with *Bradyrhizobium* species enable soybeans to produce all their nitrogen requirements while enhancing phosphorus acquisition and drought tolerance. Additionally, species in the genus *Trichoderma* inhibit pathogens while promoting induced systemic resistance in other plants, and actinobacteria such as *Streptomyces* enhance decomposition while producing antibiotics. Finally, phytohormone‐producing plant growth‐promoting rhizobacteria (PGPR), such as species in the genera Bacillus and Pseudomonas, confer resistance to abiotic stress by producing phytohormones and biofilms. Together, these beneficial relationships create a functioning microbial consortium that enhances food production while reducing the need for synthetic chemicals and enabling more reliable yields.

In conclusion, organic soybean farming systems create microbiomes that are more productive and resource‐rich than conventional farming systems. By determining the measurable effects of organic management systems on the structure of rhizosphere microbial communities, identifying the factors that guide the reorganization of the rhizosphere microbiome, and focusing on the most promising microbiome interactions for long‐term food production sustainability, this review demonstrates that the rhizosphere microbiome is a key factor in establishing a sustainable food production system. Future research may be directed toward using microbiome engineering techniques to develop organic biostimulant products tailored to specific agroecosystems, as well as identifying biomarkers for effective interaction effects across a range of environments.

## Funding

This research was funded by the International Centre for Genetic Engineering and Biotechnology (ICGEB) (Grant number: CRP/ZAF22‐93) awarded to O.O.B.

## Conflicts of Interest

The authors declare no conflicts of interest.

## Data Availability

No new data were generated. Data sharing does not apply to this article.

## References

[pei370147-bib-0002] Adedayo, A. A. , and O. O. Babalola . 2023. “Fungi That Promote Plant Growth in the Rhizosphere Boost Crop Growth.” Journal of Fungi 9, no. 239: 239. 36836352 10.3390/jof9020239 PMC9966197

[pei370147-bib-0003] Agbodjato, N. A. , and O. O. Babalola . 2024. “Promoting Sustainable Agriculture by Exploiting Plant Growth‐Promoting Rhizobacteria (PGPR) to Improve Maize and Cowpea Crops.” PeerJ 12: e16836. 38638155 10.7717/peerj.16836 PMC11025545

[pei370147-bib-0004] Agunbiade, V. F. , and O. O. Babalola . 2024. “Drought Stress Amelioration Attributes of Plant‐Associated Microbiome on Agricultural Plants.” Bioinformatics and Biology Insights 18: 11779322241233442. 38464334 10.1177/11779322241233442 PMC10924568

[pei370147-bib-0005] Agyekum, D. V. , T. Kobayashi , K. M. Dastogeer , et al. 2023. “Diversity and Function of Soybean Rhizosphere Microbiome Under Nature Farming.” Frontiers in Microbiology 14: 1130969. 36937301 10.3389/fmicb.2023.1130969 PMC10014912

[pei370147-bib-0006] Ajiboye, T. T. , A. S. Ayangbenro , and O. O. Babalola . 2022. “Functional Diversity of Microbial Communities in the Soybean ( *Glycine max* L.) Rhizosphere From Free State, South Africa.” International Journal of Molecular Sciences 23: 9422. 36012686 10.3390/ijms23169422 PMC9409019

[pei370147-bib-0007] Akanmu, A. O. , M. S. Ayilara , and O. O. Babalola . 2025. “Symbiotic Strategies for Plant Stress Tolerance: The Role of Beneficial Microbes and Omics in Sustainable Agriculture.” In Ecofriendly Frontiers: Harnessing Microbial Applications for Food Security, 189–213. Springer Nature Switzerland.

[pei370147-bib-0008] Akanmu, A. O. , O. O. Babalola , V. Venturi , et al. 2021. “Plant Disease Management: Leveraging the Plant–Microbe–Soil Interface in the Biorational Use of Organic Amendments.” Frontiers in Plant Science 12: 700507. 34394153 10.3389/fpls.2021.700507 PMC8360880

[pei370147-bib-0009] Akanmu, A. O. , O. M. Olowe , A. T. Phiri , et al. 2023. “Bioresources in Organic Farming: Implications for Sustainable Agricultural Systems.” Horticulturae 9, no. 6: 659.

[pei370147-bib-0010] Akanmu, A. O. , A. A. Sobowale , M. A. Abiala , O. J. Olawuyi , and A. C. Odebode . 2020. “Efficacy of Biochar in the Management of *Fusarium verticillioides* Sacc. Causing Ear Rot in *Zea mays* L.” Biotechnology Reports 26: e00474. 32477901 10.1016/j.btre.2020.e00474 PMC7248655

[pei370147-bib-0011] Akter, S. , M. Kamruzzaman , M. P. Sarder , et al. 2024. “Mycorrhizal Fungi Increase Plant Nutrient Uptake, Aggregate Stability and Microbial Biomass in the Clay Soil.” Symbiosis 93, no. 2: 163–176.

[pei370147-bib-0012] Almeida, O. A. C. , N. O. de Araujo , B. H. S. Dias , et al. 2023. “The Power of the Smallest: The Inhibitory Activity of Microbial Volatile Organic Compounds Against Phytopathogens.” Frontiers in Microbiology 13: 951130. 36687575 10.3389/fmicb.2022.951130 PMC9845590

[pei370147-bib-0013] Alori, E. T. , and O. O. Babalola . 2018. “Microbial Inoculants for Improving Crop Quality and Human Health in Africa.” Frontiers in Microbiology 9: 2213. 30283427 10.3389/fmicb.2018.02213 PMC6156547

[pei370147-bib-0014] Alori, E. T. , O. O. Osemwegie , A. L. Ibaba , et al. 2024. “The Importance of Soil Microorganisms in Regulating Soil Health.” Communications in Soil Science and Plant Analysis 55: 1–15.

[pei370147-bib-0015] Bai, Z. , T. Caspari , M. R. Gonzalez , et al. 2018. “Effects of Agricultural Management Practices on Soil Quality: A Review of Long‐Term Experiments for Europe and China.” Agriculture, Ecosystems & Environment 265: 1–7.

[pei370147-bib-0016] Barbieri, P. , T. Starck , A.‐S. Voisin , and T. Nesme . 2023. “Biological Nitrogen Fixation of Legumes Crops Under Organic Farming as Driven by Cropping Management: A Review.” Agricultural Systems 205: 103579.

[pei370147-bib-0017] Beillouin, D. , R. Cardinael , D. Berre , et al. 2022. “A Global Overview of Studies About Land Management, Land‐Use Change, and Climate Change Effects on Soil Organic Carbon.” Global Change Biology 28, no. 4: 1690–1702. 34873793 10.1111/gcb.15998

[pei370147-bib-0051] Bezboruah, M. , S. K. Sharma , T. Laxman , et al. 2024. “Conservation Tillage Practices and Their Role in Sustainable Farming Systems.” Journal of Experimental Agriculture International 46, no. 9: 946–959.

[pei370147-bib-0018] Bisht, N. , T. Singh , M. M. Ansari , H. Joshi , S. K. Mishra , and P. S. Chauhan . 2025. “Plant Growth‐Promoting *Bacillus amyloliquefaciens* Orchestrate Homeostasis Under Nutrient Deficiency Exacerbated Drought and Salinity Stress in *Oryza sativa* L. Seedlings.” Planta 261, no. 1: 8. 10.1007/s00425-024-04585-x 39643822

[pei370147-bib-0054] Byers, A. K. , S. A. Wakelin , L. Condron , and A. Black . 2024. “Land Use Change Disrupts the Network Complexity and Stability of Soil Microbial Carbon Cycling Genes Across an Agricultural Mosaic Landscape.” Microbial Ecology 87, no. 1: 167. 10.1007/s00248-024-02487-9 PMC11706911 39777550

[pei370147-bib-0056] Ciuffreda, P. , O. Xynomilakis , S. Casati , and R. Ottria . 2024. “Fluorescence‐Based Enzyme Activity Assay: Ascertaining the Activity and Inhibition of Endocannabinoid Hydrolytic Enzymes.” International Journal of Molecular Sciences 25, no. 14: 7693. 39062935 10.3390/ijms25147693 PMC11276806

[pei370147-bib-0019] Crystal‐Ornelas, R. , R. Thapa , and K. L. Tully . 2021. “Soil Organic Carbon Is Affected by Organic Amendments, Conservation Tillage, and Cover Cropping in Organic Farming Systems: A Meta‐Analysis.” Agriculture, Ecosystems & Environment 312: 107356.

[pei370147-bib-0057] Dlamini, S. P. , A. O. Akanmu , and O. O. Babalola . 2022. “Rhizospheric Microorganisms: The Gateway to a Sustainable Plant Health.” Frontiers in Sustainable Food Systems 6: 925802.

[pei370147-bib-0020] Fadiji, A. E. , A. N. Yadav , G. Santoyo , and O. O. Babalola . 2023. “Understanding the Plant–Microbe Interactions in Environments Exposed to Abiotic Stresses: An Overview.” Microbiological Research 271: 127368. 36965460 10.1016/j.micres.2023.127368

[pei370147-bib-0021] Fasusi, O. A. , O. O. Babalola , and T. O. Adejumo . 2023. “Harnessing of Plant Growth‐Promoting Rhizobacteria and Arbuscular Mycorrhizal Fungi in Agroecosystem Sustainability.” CABI Agriculture and Bioscience 4: 26.

[pei370147-bib-0022] Fontaine, S. , L. Abbadie , M. Aubert , et al. 2024. “Plant–Soil Synchrony in Nutrient Cycles: Learning From Ecosystems to Design Sustainable Agrosystems.” Global Change Biology 30, no. 1: e17034. 38273527 10.1111/gcb.17034

[pei370147-bib-0023] Fornes, F. , A. Lidón , R. M. Belda , et al. 2024. “Soil Fertility and Plant Nutrition in an Organic Olive Orchard After 5 Years of Amendment With Compost, Biochar or Their Blend.” Scientific Reports 14: 16606. 39025936 10.1038/s41598-024-67565-x PMC11258141

[pei370147-bib-0055] Galeano, R. M. S. , J. V. S. Ribeiro , S. M. Silva , et al. 2024. “New Strains of Trichoderma With Potential for Biocontrol and Plant Growth Promotion Improve Early Soybean Growth and Development.” Journal of Plant Growth Regulation 43, no. 11: 4099–4119.

[pei370147-bib-0060] Hallett, P. D. , M. Marin , G. D. Bending , T. S. George , C. D. Collins , and W. Otten . 2022. “Building Soil Sustainability From Root–Soil Interface Traits.” Trends in Plant Science 27, no. 7: 688–698. 35168900 10.1016/j.tplants.2022.01.010

[pei370147-bib-0024] Haskett, T. L. , R. Karunakaran , M. Bueno Batista , R. Dixon , and P. S. Poole . 2022. “Control of Nitrogen Fixation and Ammonia Excretion in *Azorhizobium caulinodans* .” PLoS Genetics 18: e1010276. 35727841 10.1371/journal.pgen.1010276 PMC9249168

[pei370147-bib-0025] Igiehon, B. C. , O. O. Babalola , and A. I. Hassen . 2024. “Rhizosphere Competence and Applications of Plant Growth‐Promoting Rhizobacteria in Food Production: A Review.” Scientific African 23: e02081.

[pei370147-bib-0026] Ishfaq, M. , Y. Wang , J. Xu , et al. 2023. “Improvement of Nutritional Quality of Food Crops With Fertilizer: A Global Meta‐Analysis.” Agronomy for Sustainable Development 43, no. 6: 74.

[pei370147-bib-0027] Joergensen, R. G. , M. Hemkemeyer , L. Beule , et al. 2024. “A Hitchhiker's Guide: Estimates of Microbial Biomass and Microbial Gene Abundance in Soil.” Biology and Fertility of Soils 60: 457–470.

[pei370147-bib-0028] Jung, J. , S. Ahn , D.‐H. Kim , and M. Riu . 2024. “Triple Interactions for Induced Systemic Resistance in Plants.” Frontiers in Plant Science 15: 1464710. 39649811 10.3389/fpls.2024.1464710 PMC11620860

[pei370147-bib-0050] Kabir, Z. , R. Singh , and R. Ghimire . 2023. “Soil pH Regulation and Legume–Rhizobia Symbiosis.” Plant and Soil 485, no. 1–2: 215–230.

[pei370147-bib-0029] Khan, A. , A. V. Singh , S. S. Gautam , et al. 2023. “Microbial Bioformulation: A Microbial Assisted Biostimulating Fertilization Technique for Sustainable Agriculture.” Frontiers in Plant Science 14: 1270039. 38148858 10.3389/fpls.2023.1270039 PMC10749938

[pei370147-bib-0063] Kuzyakov, Y. , and B. S. Razavi . 2019. “Rhizosphere Size and Shape: Temporal Dynamics and Spatial Stationarity.” Soil Biology and Biochemistry 135: 343–360.

[pei370147-bib-0030] Li, H. , G. Liu , C. Dan , et al. 2025. “Effects of Soil Porosity on Water Stability of Aggregates.” Soil & Tillage Research 254: 106741.

[pei370147-bib-0031] Li, W.‐J. , X.‐Y. Zhou , X.‐L. An , et al. 2024. “Enhancement of Beneficial Microbiomes in Plant–Soil Continuums Through Organic Fertilization: Insights Into the Composition and Multifunctionality.” Soil Ecology Letters 6: 230223.

[pei370147-bib-0032] Liu, J. , A. Shu , W. Song , et al. 2021. “Long‐Term Organic Fertilizer Substitution Increases Rice Yield by Improving Soil Properties and Regulating Soil Bacteria.” Geoderma 404: 115287.

[pei370147-bib-0033] Liu, X. , Q. Chen , H. Zhang , et al. 2023. “Effects of Exogenous Organic Matter Addition on Agricultural Soil Microbial Communities and Relevant Enzyme Activities in Southern China.” Scientific Reports 13, no. 1: 8045. 37198213 10.1038/s41598-023-33498-0 PMC10192384

[pei370147-bib-0034] Liu, Y. , A. Shi , Y. Chen , et al. 2024. “Beneficial Microorganisms: Regulating Growth and Defense for Plant Welfare.” Plant Biotechnology Journal 23: 986–998. 39704146 10.1111/pbi.14554 PMC11869181

[pei370147-bib-0059] Mao, J. , Q. Mao , P. Gundersen , et al. 2022. “Unexpected High Retention of ^15^N‐Labeled Nitrogen in a Tropical Legume Forest Under Long‐Term Nitrogen Enrichment.” Global Change Biology 28, no. 4: 1529–1543. 34800306 10.1111/gcb.16005

[pei370147-bib-0035] Mátyás, B. , M. E. C. Andrade , N. C. Y. Chida , et al. 2018. “Comparing Organic Versus Conventional Soil Management on Soil Respiration.” F1000Research 7: 258. 29623193 10.12688/f1000research.13852.1 PMC5861514

[pei370147-bib-0061] Migliore, G. , G. Rizzo , A. Bonanno , E. C. Dudinskaya , J. Tóth , and G. Schifani . 2022. “Functional Food Characteristics in Organic Food Products—The Perspectives of Italian Consumers on Organic Eggs Enriched With Omega‐3 Polyunsaturated Fatty Acids.” Organic Agriculture 12, no. 2: 149–161.

[pei370147-bib-0036] Mishra, A. K. , P. Yadav , S. Sharma , and P. Maurya . 2025. “Comparison of Microbial Diversity and Community Structure in Soils Managed With Organic and Chemical Fertilization Strategies Using Amplicon Sequencing.” Frontiers in Microbiology 15: 1444903. 40017465 10.3389/fmicb.2024.1444903 PMC11865238

[pei370147-bib-0037] Moretti, L. G. , C. A. C. Crusciol , M. F. A. Leite , et al. 2024. “Diverse Bacterial Consortia: Key Drivers of Rhizosoil Fertility Modulating Microbiome Functions, Plant Physiology, Nutrition, and Soybean Grain Yield.” Environmental Microbiomes 19: 50. 10.1186/s40793-024-00595-0 PMC11264919 39030648

[pei370147-bib-0062] Olanrewaju, O. S. , A. S. Ayangbenro , B. R. Glick , and O. O. Babalola . 2019. “Plant Health: Feedback Effect of Root Exudates‐Rhizobiome Interactions.” Applied Microbiology and Biotechnology 103, no. 3: 1155–1166. 30570692 10.1007/s00253-018-9556-6 PMC6394481

[pei370147-bib-0053] Oliveira, F. C. , G. W. Ferreira , S. J. Assuncao , and A. Pedrotti . 2024. “Long‐Term Impacts of Tillage and Cover Cropping on Soil Organic Carbon and Carbon Oxidizable Fractions in a Tropical Sandy Soil.” Journal of Soil Science and Plant Nutrition 24, no. 4: 7640–7650.

[pei370147-bib-0038] Pedrinho, A. , L. W. Mendes , A. P. de Araujo Pereira , et al. 2024. “Soil Microbial Diversity Plays an Important Role in Resisting and Restoring Degraded Ecosystems.” Plant and Soil 500: 325–349.

[pei370147-bib-0039] Peng, J. , Y. Wu , T. Geng , C. Zhang , J. Wang , and C. Cai . 2024. “Soil Pore Dynamics and Infiltration Characteristics as Affected by Cultivation Duration for Mollisol in Northeast China.” Geoderma 449: 117021.

[pei370147-bib-0040] Rashid, H.‐o. , M. Krehenbrink , and M. S. Akhtar . 2014. “Nitrogen‐Fixing Plant‐Microbe Symbioses.” In Sustainable Agriculture Reviews 15, 193–234. *Springer* .

[pei370147-bib-0058] Rieke, E. L. , S. B. Cappellazzi , M. Cope , et al. 2022. “Linking Soil Microbial Community Structure to Potential Carbon Mineralization: A Continental Scale Assessment of Reduced Tillage.” Soil Biology and Biochemistry 168: 108618.

[pei370147-bib-0052] Sarker, M. R. , M. V. Galdos , A. J. Challinor , M. S. Huda , A. K. Chaki , and A. Hossain . 2022. “Conservation Tillage and Residue Management Improve Soil Health and Crop Productivity—Evidence From a Rice‐Maize Cropping System in Bangladesh.” Frontiers in Environmental Science 10: 969819.

[pei370147-bib-0041] Sharma, A. , X. Wu , and A. L. Khan . 2024a. “Plant Growth‐Promoting Bacteria Enhance Soybean Resilience to Drought and Salinity Stress.” Plant Physiology and Biochemistry 201: 107845.

[pei370147-bib-0042] Sharma, V. , D. Sharma , and R. Salwan . 2024b. “Surviving the Stress: Understanding the Molecular Basis of Plant Adaptations and Uncovering the Role of Mycorrhizal Association in Plant Abiotic Stresses.” Microbial Pathogenesis 193: 106772. 38969183 10.1016/j.micpath.2024.106772

[pei370147-bib-0043] Smith, P. , J. I. House , M. Bustamante , et al. 2016. “Global Change Pressures on Soils From Land Use and Management.” Global Change Biology 22, no. 3: 1008–1028. 26301476 10.1111/gcb.13068

[pei370147-bib-0044] Toth, M. , C. Stumpp , A. Klik , et al. 2024. “Long‐Term Effects of Tillage Systems on Soil Health of a Silt Loam in Lower Austria.” Soil and Tillage Research 241: 106120.

[pei370147-bib-0045] Uwituze, Y. , J. Nyiraneza , T. D. Fraser , J. Dessureaut‐Rompré , N. Ziadi , and J. Lafond . 2022. “Carbon, Nitrogen, Phosphorus, and Extracellular Soil Enzyme Responses to Different Land Use.” Frontiers in Soil Science 2: 814554.

[pei370147-bib-0046] Uzoh, I. M. , K. C. Ramadile , U. P. Chukwudi , O. N. Igiehon , A. O. Akanmu , and O. O. Babalola . 2025. “Biochar Soil Amendments Affect Mycorrhizal Colonization, Root Nodulation and Dry Matter Accumulation in Cowpeas ( *Vigna unguiculata* (L.) Walp.).” Studia Universitatis Babeş‐Bolyai, Biologia 70, no. 1: 153–172.

[pei370147-bib-0047] Wu, Z. , X. Chen , X. Lu , et al. 2024. “Impact of Combined Organic Amendments and Chemical Fertilizers on Soil Microbial Limitations, Soil Quality, and Soybean Yield.” Plant and Soil 507, no. 1: 317–334.

[pei370147-bib-0048] Zhang, Z. , D. Xie , W. Teng , et al. 2025. “A State of Art Review on Carbon, Nitrogen, and Phosphorus Cycling and Efficient Utilization in Paddy Fields.” Plant and Soil 513, no. 2: 1689–1709.

[pei370147-bib-0049] Zhao, Y.‐Y. , H.‐J. Jiang , F.‐J. Xu , et al. 2022. “Soil Acidification Negatively Affects Arachis Hypogeae L. Growth by Inhibiting Nodule Initiation and Nitrogen Fixation.” Journal of Soil Science and Plant Nutrition 22, no. 1: 571–584.

